# Educational Video Intervention to Improve Health Misinformation Identification on WhatsApp Among Saudi Arabian Population: Pre-Post Intervention Study

**DOI:** 10.2196/50211

**Published:** 2024-01-17

**Authors:** Ebtihal Alsaad, Sharifah AlDossary

**Affiliations:** 1 Department of Health Informatics College of Public Health and Health Informatics King Saud bin Abdulaziz University for Health Sciences Riyadh Saudi Arabia; 2 King Abdullah International Medical Research Center Riyadh Saudi Arabia

**Keywords:** misinformation, education, WhatsApp, intervention, pre-postintervention design, health literacy, educational, video, videos, consumer, consumers, patient education, survey, surveys, web-based information, health information, reliability, accuracy, reliable, social media

## Abstract

**Background:**

Health misinformation can adversely affect individuals’ quality of life and increase the risk of mortality. People often fail to assess the content of messages before sharing them on the internet, increasing the spread of misinformation. The problem is exacerbated by the growing variety of digital information environments, especially social media, which presents as an effective platform for spreading misinformation due to its rapid information-sharing capabilities. Educational interventions have been developed to help consumers verify the validity of digital health information. However, tools designed to detect health misinformation on social media content have not been validated. Given the increased use of social media platforms, particularly WhatsApp, it is crucial to develop tools to help consumers assess the credibility of messages and detect misinformation.

**Objective:**

The main objective of this study is to develop and assess an educational tool aimed at educating consumers about detecting health misinformation on WhatsApp. The secondary objective is to assess the association between demographic factors and knowledge levels.

**Methods:**

The study used a single-arm, pre-post intervention design to evaluate the effectiveness of an educational video in improving participants’ ability to detect health-related misinformation in WhatsApp messages. In the first phase, an educational video intervention was developed and validated. In the second phase, participants were invited to complete a web-based survey that consisted of pre-evaluation questions, followed by the educational video intervention. Subsequently, they were asked to answer the same questions as the postevaluation questions.

**Results:**

The web-based survey received 485 responses. The completion rate was 99.6% (n=483). Statistically significant associations existed between knowledge level and age, gender, employment, and region of residence (*P*<.05). The video intervention did elicit a statistically significant change in the participants’ abilities to identify misinformation in WhatsApp messages (*z*=–6.887; *P*<.001). Viewing the video was associated with increased knowledge about the following concepts: checking the “forwarded” label (*P*<.001), looking for spelling and grammatical errors (*P*<.001), analyzing the facts (*P*=.03), checking links (*P*=.002, *P*=.001), and assessing the photos and videos (*P*<.001). There was a statistically significant difference in knowledge level before and after the intervention (*P*<.001).

**Conclusions:**

This study developed and evaluated the effectiveness of an educational video intervention to improve health misinformation identification on WhatsApp among the Saudi Arabian population. The results indicate that educational videos can be valuable tools for improving participants’ abilities to identify misinformation. The outcomes of this research can contribute to our understanding of what constitutes an effective tool for enhancing health misinformation awareness. Such interventions may be particularly useful in combating misinformation among Arabic-speaking populations on WhatsApp, which may ultimately improve eHealth literacy. Limiting the prevalence and impact of misinformation allows people to make better-informed health decisions.

## Introduction

### Background

Researching health problems and learning about health via the internet has become a prevalent practice [1]. The level of credibility of this health-related information and the way it is used by patients, caregivers, and other health consumers have garnered the attention of health care providers and authorities [2]. There are many inaccurate sources of information on the internet, and this can lead to users becoming misinformed. According to Chou et al [3], health misinformation is defined as any health-related factual claim that is false according to recent scientific evidence. Misinformation about health can adversely affect quality of life and even increase one’s mortality risk [1].

When the COVID-19 pandemic first started, the amount of information related to this new global pandemic increased at an unprecedented rate. The volume of information, as well as the rate at which new information appeared, increased rapidly [4]. Global pandemics such as COVID-19 are likely to lead to the increased spread of misinformation as people explore massive amounts of information about the disease and its health implications. The term “infodemic” is used to describe the current media environment, which is characterized by an overflow of both true and false information. During the pandemic, individuals generally look for accurate, unbiased information, but these sources may be hidden among misinformation spread through the infodemic [5].

Due to its capacity to rapidly disseminate information, social media can serve as a platform for the propagation of misinformation. The abundance of available information can lead to the predominance of misinformation, thus negatively affecting cognitive, logical, and decision-making capacities. WhatsApp, Twitter, and Facebook are the most commonly used social media platforms for spreading false information. Since the beginning of the COVID-19 pandemic, internet use has expanded worldwide, which has resulted in the proliferation of incorrect information via social media [6].

Saudi Arabia, with a population of over 35 million, is the second largest Arab country [7]. The COVID-19 pandemic has had a significant impact on internet usage in Saudi Arabia, with a reported increase to 91.2% (n=28,775,889) in 2020. This represents a rise of 2.6 percentage points compared to the previous year [8]. Alshareef and Alotiby [6] used a web-based survey to investigate the most widely used social media platforms in Saudi Arabia, the proportions of Saudi Arabians who used these platforms to share information, and these users’ perceptions of the medical information shared on these platforms. According to their survey results, WhatsApp was used by 52.4% (n=144) of health care workers and 51.3% (n=500) of non–health care workers to circulate information. The findings of their study concluded that WhatsApp is the most commonly used social network among Saudi Arabians. COVID-19–related information is, therefore, more likely to be shared on this application [6].

Another study by Alasmari et al [9] found that social media platforms, with their capacity to quickly disseminate information, comprised the primary source of falsehoods spread in the community. Based on an examination of the social media platforms, the study revealed that WhatsApp users accounted for approximately 46% (n=41) of rumor sources on the internet in Saudi Arabia.

Additionally, research by Tan et al [10] examined daily WhatsApp use for receiving, forwarding, or discussing COVID-19–related content a in 1-week period. The results indicate that almost every respondent participated in conversations about COVID-19. However, users were more likely to share or receive forwarded messages than to engage in active, original conversations about COVID-19. A high volume of forwarded messages was observed; this is concerning because the developers of WhatsApp have linked forwarded messages with misinformation.

People rarely assess the content of messages before sharing them on social media platforms, and they frequently fail to verify whether the messages are accurate. Educating consumers about identifying misinformation and dealing with the infodemic is essential. The false information epidemic compromises public health as misinformation spreads throughout social media. It is critical to increase awareness about the nature of social media and how to use it effectively. Personal responsibility is the first and most crucial step in safeguarding our community from the harmful phenomena of misinformation [11].

To effectively access health-related information on the internet, consumers must be able to assess the quality of the information that they find. This is a crucial aspect of eHealth literacy. It remains difficult for digital health consumers to determine the quality of the information placed in front of them. The problem becomes more complex as the digital information environment becomes more complicated and heterogeneous, especially with the rise of social media, where anyone can spread information about health and where low-quality and misleading information spreads rapidly. Interventions are urgently needed to address this public health problem [12].

Several interventions have been developed to assist consumers in verifying the validity of digital health information [12]. A systematic review by Cusack et al [13] examined studies on educational interventions that aimed to improve knowledge of essential concepts, enabling health interventions to be evaluated for their impacts. According to the study, educational interventions, at least in the short term, can increase people’s knowledge and skills in evaluating health claims.

For the detection of health misinformation, interventions have been established based on instruments that allow anyone, including those with no prior medical background, to differentiate fact from fiction. However, these tools were designed for lengthy texts (such as text found on websites) and have not yet been validated for detecting health misinformation in social media content [14]. The majority of the tools developed were used to assess the quality of websites that provided health information. Considering the increased use of social media platforms—primarily WhatsApp—in Saudi Arabia for sharing health information, it is essential to develop tools that help consumers assess the credibility of messages and detect misinformation.

The first World Health Organization Infodemiology conference for managing the infodemic suggested evidence-based analysis and interventions to reduce the harmful effects of health misinformation during acute health events. Among the recommendations was the development of interventions that address factors that impact trust and resilience to misinformation at the individual, community, cultural, and societal levels [15].

### Theoretical Background

A low level of health literacy has been recognized as one of the factors contributing to the infodemic. Other contributing factors include the widespread use of social media, quick publication processes, and preprint services. Rumor-spreading behavior also plays a role in the infodemic as do anxiety, distress, and fear [16]. According to a systematic review by Diviani et al [17], health literacy is essential when evaluating digital health information. Individuals’ abilities to find, evaluate, and use health information empowers them to actively deal with the misinformation they encounter on social media. In order to prevent people from automatically accepting health rumors as facts, health literacy must be improved [18].

In this research, the educational intervention concept was guided by the inoculation theory and the message interpretation process theory. According to the inoculation theory, previous experience helps individuals combat future attacks [19]. For example, literacy interventions may help audiences resist harmful media messages by providing them with the knowledge and skills necessary to reject them [20]. Based on the message interpretation process theory, exposure to message interventions influences subsequent decision-making when dealing with harmful information [21]. Both theories identify the role of an intervention or prior messages in influencing the cause of action [22].

The choice of the intervention media was guided by the cognitive theory of multimedia learning, which is built from the cognitive load theory and states that working memory contains 2 channels for acquiring and processing information an auditory or verbal channel and a visual or pictorial channel. Although each channel has a limited capacity, the 2 can be used together to integrate new information more easily. Working memory can function at its best when both channels are used. However, 1 or both channels can become overloaded by a heavy cognitive load. It is possible to improve learning through the use of multimedia learning materials that manage the cognitive load across both channels. Furthermore, the cognitive theory of multimedia learning states that any learning should involve cognitive processing to be meaningful. Cognitive processing requires a learner to pay attention to the material presented, organize it mentally, and integrate it into prior knowledge [23].

### Objectives

The main objective of this study is to develop and assess an educational tool aimed at educating consumers about detecting health misinformation on WhatsApp. The secondary objective is to assess the association between demographic factors and knowledge levels.

## Methods

### Study Design

The study used a single-arm, pre-post intervention design to evaluate the effectiveness of educational video in improving participants’ ability to detect health-related misinformation in WhatsApp messages. The study’s first phase was developing and validating an educational video intervention. In the second phase, participants were invited to complete a web-based survey that contained pre-evaluation questions, the intervention, and postevaluation questions.

### Participants

A web-based survey was distributed among the general Saudi population from November 24 to December 25, 2022. The survey was disseminated through social media networks (WhatsApp, Instagram, Twitter, Facebook, and Telegram), and the data were collected using Google Forms.

It has been estimated that 82% (n=29.50 million) of Saudis use social networks daily, with varying usage rates among different platforms. Among these platforms, WhatsApp is the most widely used social network with 87.4% (n=30.67 million) of internet users in Saudi Arabia, followed by Instagram (n=27.40 million, 78.1%), Twitter (n=25.23 million, 71.9%), Facebook (n=22.25 million, 63.4%), and Telegram (n=20.88 million, 59.5%) [24]. In order to target a wide range of the population, the web-based survey was disseminated across all of these social media platforms.

The study population consisted of social media users in the general population of the Kingdom of Saudi Arabia. The inclusion criteria were (1) having the ability to complete an anonymous survey questionnaire on the internet, (2) being at least 18 years of age, and (3) understanding Arabic.

The sample size was calculated using the Raosoft sample size calculator, based on the total population of Saudi Arabia (n=35,013,414), with a 95% CI [7,25]. This calculation yielded a minimum sample size of 385 using absolute error or precision of 0.05. This sample size is sufficient to detect a difference between pre and postscore with an effect size of 0.15 (small effect size) using a power of 80% and α of .05.

The study population was targeted using a convenience sampling technique with no predetermined sampling frame. Convenience sampling is a nonprobability method in which individuals are sampled simply because they are “convenient” data sources [26].

Several specific methods were used for the recruitment process. First, as there are social accounts run by the public to share news and announcements related to each region in Saudi Arabia, the survey was distributed to these public social networking groups on various social media platforms. Second, the researchers approached social media influencers on different platforms to spread the survey to more participants. Third, the researchers asked all their social media contacts to consider completing the survey and sharing it with their contacts on social networks.

The web-based survey had 4 sections. In the first section, the participants were asked to provide demographic information. In the second section, they were given a set of pretest questions asking them to identify whether a WhatsApp message contained correct or false information. In the third section, the participants were shown an educational video. After finishing the video, the participants moved to the last section, which contained the same set of questions as the pretest.

There were no records of participant identity, and confidentiality was ensured. Upon completion of the survey, a message of thanks appeared. No incentives were offered for completing the survey.

### Intervention (Educational Video)

#### Educational Video Design

This study used a short video intervention. The content of the educational video was developed based on three sources: (1) the recommendations on WhatsApp’s official website regarding how to prevent the spread of misinformation; (2) the World Health Organization’s (WHO’s) advice on how to navigate the infodemic and identify misinformation; and (3) the CRAAP test, a tool for evaluating the quality of a social media source by assessing its currency, relevance, authority, accuracy, and purpose [27-29]. The educational tool introduced 6 concepts that could be used to assess and identify misinformation in WhatsApp messages. These concepts included checking the “forwarded” label, looking for spelling and grammatical errors, reading beyond the headline, analyzing the facts, checking links, and assessing photos and videos (Table 1).

**Table 1:** Concepts used in the video to evaluate and identify misinformation in WhatsApp messages.

Concept	Source
Check the “forwarded” label	WhatsApp [27]
Look for spelling and grammatical errors	WHO^a^, WhatsApp, and CRAAP Test [27-29]
Read beyond the headline	WHO [28]
Analyze the facts	WHO, WhatsApp, and CRAAP Test [27-29]
Check links	CRAAP Test [29]
Assess the photos and videos	WHO [28]

^a^WHO: World Health Organization.

**Figure 1:**
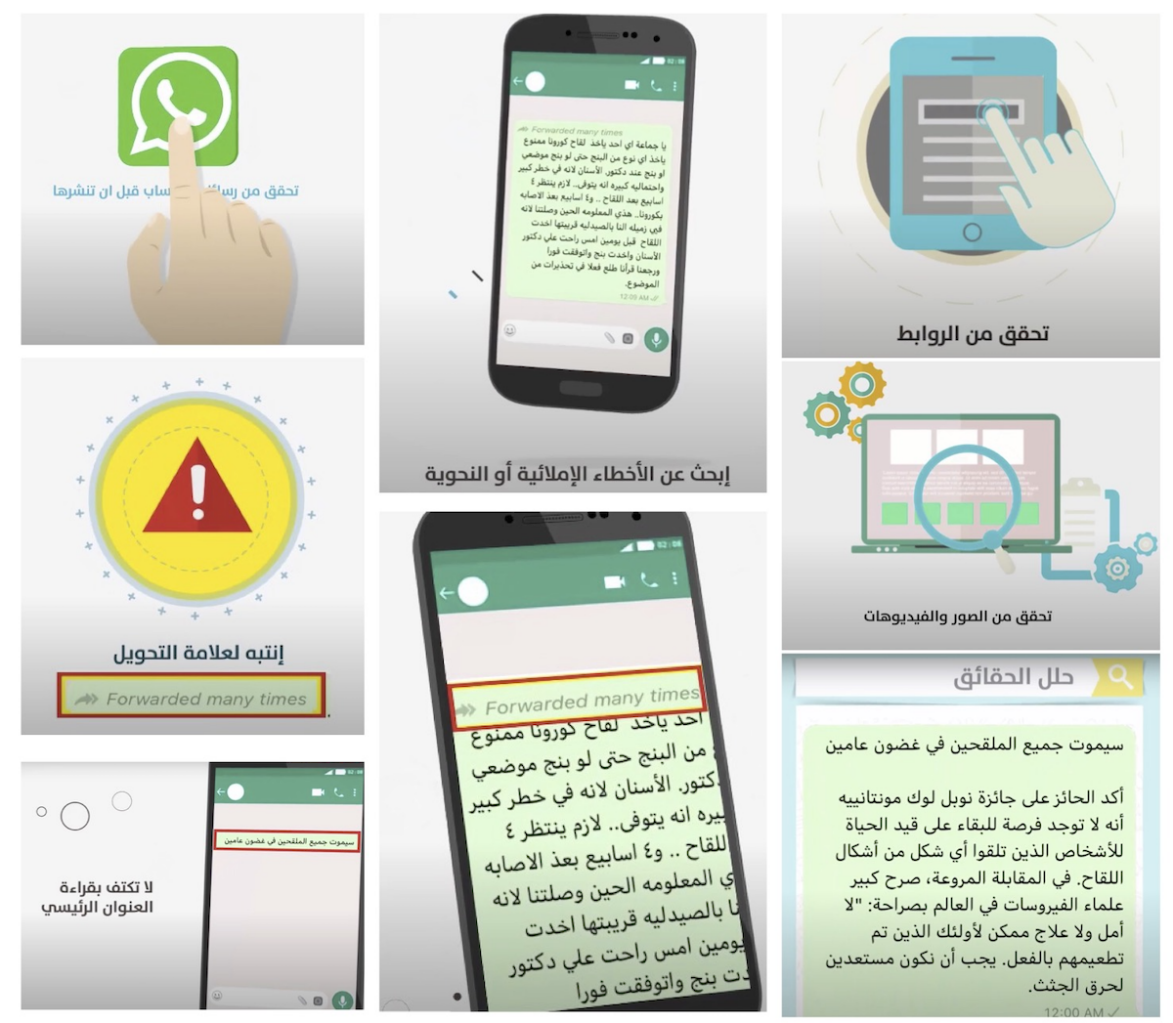
Screenshots from the educational video.

The design of the educational video was based on literature guidelines for the design of health education messages [30]. Following Hugo recommendation, the construction of the educational material included the consideration of communication principles and sociocultural factors, including the literacy levels and language preferences of the audience, to design appropriate messages. When designing the audiovisual content, simplicity (text and visual composition) and the audience’s emotional involvement were considered [30]. The educational video was developed in classical Arabic to make it accessible to a wider audience. Multimedia Appendix 1 shows the developed educational video, while screenshots of the educational video are shown in Figure 1.

#### Educational Video Validation Process

Yusoff [31] recommended that when validating the content of a tool, a minimum of 6 (but no more than 10) experts should be involved in the assessment process. The validation of the educational material included assessments by 7 experts (Multimedia Appendix 2). For this study, it was essential that the specialists were well-versed in the Arabic language (spoken and written).

To identify the broad range of expertise needed, roles were categorized into different fields. Four roles were identified: (1) health informatics experts, (2) health education specialists, (3) infodemic managers, and (4) public health experts.

Each category was populated with a representative through purposeful sampling. A panel of experts was formed with at least 1 representative from each role; email was used to contact the representatives and provide information about the study. The validation forms and the educational video were delivered to the specialists via email.

The validation form was created by combining 2 tools from which items relevant to the study were selected. Questions Q2 through Q11 were adopted from the Educational Content Validation Instrument in Health developed by Leite et al [32], and questions Q1 and Q12 through Q17 were adopted from an audiovisual content evaluation instrument constructed by Rosa et al [33].

The final evaluation form contained 17 questions covering three areas: objectives, structure and presentation, and audiovisuals (Multimedia Appendix 3). The “objectives” section focused on purposes and goals, whereas the “structure and presentation” section emphasized organization, structure, strategy, sufficiency, and consistency. As for the “audiovisual” area, the emphasis was on the technological aspect. A score of 0 indicated disagreement, 1 indicated partial agreement, and 2 indicated strong agreement with the value of the items [34].

#### Educational Video Validation Result

A content validity index was used to analyze the results. Content validity indexes can be computed in 2 ways. One type of validity is item-level content validity indexes (I-CVIs), which consider the content validity of individual items. The other type is scale-level content validity indexes, which involve a scale’s overall content validity [35]. For the scale-level content validity, calculations were conducted using the scale-level content validity index averaging method (S-CVI/Ave) as recommended by Polit and Beck [36].

The calculations were carried out manually. The items ranked “disagree” were scored as 0, whereas the items ranked “partially agree” and “strongly agree” were scored as 1 [34].

To obtain excellent content validity, the content of educational videos must have items with I-CVIs above 0.78 for (6 to 10 experts) and an S-CVI/Ave of 0.90 or higher [36]. When the I-CVI is below 0.78 and the S-CVI/Ave is below 0.90, the content modification should be considered for that particular educational video area.

As shown in Table 2, all items had I-CVIs greater than 0.78 (78%), indicating agreement between the experts’ answers. In terms of scale evaluation, all the 3 areas (objectives, structure and presentation, and audiovisual) had S-CVI/Aves above 0.90 (90%). In the objectives area, item 5 (“stimulates interest in the theme”) had the lowest specialist agreement score (6 out of 7 or an I-CVI of 0.86). The overall S-CVI/Ave for the objectives area was 0.97.

**Table 2:** A content validity index calculation using 7 expert ratings to validate the video’s content.

Item			Expert 1		Expert 2		Expert 3		Expert 4	Expert 5		Expert 6		Expert 7		Experts In Agreement		I-CVI^a^
**Objectives^b^**																		
	Q1	1		1		1		1		1	1		1		7		1	
	Q2	1		1		1		1		1	1		1		7		1	
	Q3	1		1		1		1		1	1		1		7		1	
	Q4	1		1		1		1		1	1		1		7		1	
	Q5	1		1		1		1		1	0		1		6		0.86	
	Proportion relevance	1		1		1		1		1	0.8		1					
**Structure and presentation^c^**																		
	Q6	1		1		1		1		1	1		1		7		1	
	Q7	1		1		1		1		1	1		1		7		1	
	Q8	1		1		1		1		1	1		1		7		1	
	Q9	1		1		1		1		1	1		1		7		1	
	Q10	1		1		1		1		1	1		1		7		1	
	Q11	1		1		1		1		1	1		1		7		1	
	Q12	1		1		1		1		1	1		1		7		1	
	Proportion relevance	1		1		1		1		1	1		1					
**Audiovisual^d^**																		
	Q13	1		1		1		1		1	1		1		7		1	
	Q14	1		1		1		0		1	1		1		6		0.86	
	Q15	1		1		1		1		1	1		1		7		1	
	Q16	1		1		1		1		1	1		1		7		1	
	Q17	1		1		0		1		1	1		1		6		0.86	
	Proportion relevance	1		1		0.8		0.8		1	1		1					

^a^I-CVI: item-level content validity index.

^b^The S-CVI/Ave and average proportion of items judged as relevance across the 7 experts for objectives is 0.97.

^c^The S-CVI/Ave and average proportion of items judged as relevance across the 7 experts for structure and presentation is 1.

^d^The S-CVI/Ave and average proportion of items judged as relevance across the 7 experts for audiovisual is 0.94.

The structure and presentation area had the highest level of agreement (100% S-CVI/Ave). In the audiovisual area, 2 items (“the illustrations are expressive and sufficient” and “the characters/images are appropriate for the target audience”) had scores of 6 out of 7 or I-CVIs of 0.86. The overall S-CVI/Ave for the audiovisual area was 0.94.

### Data Collection Tool

#### Development of the Survey

The study aimed to develop and assess an interventional tool to educate the Saudi population on how to identify health misinformation in WhatsApp messages. The developed Arabic survey contained 4 sections: demographic data, pretest questions, educational intervention, and posttest questions.

The first section (sociodemographic information) included 7 questions about each participant’s background information, including gender, age, educational level, employment status, region of residence, city of residence, and nationality. The second section included 8 questions that assessed each participant’s ability to identify misinformation based on the WhatsApp messages evaluation concepts mentioned in Table 1. Multimedia Appendix 4 shows the complete list of questions that were assigned to the concepts. The third section included the educational tool, which discussed 6 concepts that could be used to identify misinformation in WhatsApp messages. The fourth section included the same 8 questions as the second domain to measure the effectiveness of the educational tool.

The assessment questions were based on real examples representing the different domains of WhatsApp messages. The 8 pre and posttest questions included 5 messages with misinformation (based on messages circulated during the pandemic) and 3 with correct information (obtained from the Saudi Ministry of Health’s official website) [37]. The evaluation concepts, selection of the messages, and their relevancy were assessed by 2 authors (EA and SA). EA collected the messages and placed them under each domain, and SA assessed the relevancy; any uncertainty was resolved by consensus. Finally, a summary of the study’s goal was included in the survey, as was a statement assuring the respondents’ confidentiality.

The highest possible score for the survey questions was 8 points (correct answers were scored as 1 point; incorrect answers and responses of “I do not know” were scored as 0 points). We used a modified Bloom cutoff value of 75% to categorize participants’ knowledge. As a result, we considered participants with scores ≥75% to have high knowledge. Participants with scores below 75% were considered to have low knowledge. This cutoff value is based on previous publications [38,39].

#### Pilot Survey

The web-based survey was pilot tested, and a total of 31 participants responded. During the first piloting stage, the survey was sent to 15 participants, 3 of whom commented that the instructions needed clarification. Subsequently, an instruction section containing a description of the other survey sections was added at the beginning, and the survey was distributed again to 16 participants. In the second stage, no further comments were received; all participants indicated that the survey was clear. The internal consistency of the final survey was measured using Cronbach α. The scale had a Cronbach α of .847, demonstrating good internal reliability. The final version of the survey can be found in Multimedia Appendix 5.

### Ethical Considerations

The study was approved by King Abdullah International Medical Research Center (reference RYD-22-419812-107000). The survey included a summary of the study’s purpose and a statement that, by completing the survey, the respondents agreed to participate in this research. The confidentiality of the study participants was ensured by not collecting identifiable data, encrypting files, and requiring a password to open or modify files.

### Statistical Analysis

The demographic characteristics of the participants were reported using descriptive statistics, such as frequencies and percentages. Categorical variables were analyzed using chi-square tests to determine the associations between the demographic variables and the knowledge levels of the participants. A modified Bloom cutoff was used to categorize the knowledge levels. The normality of the variables was analyzed using the Kolmogorov-Smirnov test and the Shapiro-Wilk test. The median scores from before and after the educational video were compared using the Wilcoxon signed-rank test. The McNemar test for categorical data was used to compare the answers and differences in knowledge levels before and after the educational video intervention. The analyses were performed using SPSS Statistics (version 29.0; IBM). A *P*-value of .05 or less was considered statistically significant.

## Results

### Characteristics of Study Participants

The web-based survey received 485 responses, and 2 did not agree to participate, giving a 99.6% (n=483) completion rate. In total, 483 responses were analyzed. The socioeconomic characteristics of the participants are shown in Table 3. Most of the study respondents (n=457, 94.6%) were Saudis. More than half of the participants (n=300, 62.1%) were female, and more than half were in the age range of either 18-24 or 25-34 years (n=130, 26.9% and n=173, 35.8%, respectively). More than half of the sample (n=275, 56.9%) had bachelor degrees. With regard to the employment status, 45.5% (n=220) of the respondents were employed. The highest number of participants came from the eastern region (n=181, 37.5%), followed by the central (n=132, 27.3%) and western (n=83, 17.2%) regions.

**Table 3:** Participant demographics of the full sample (N=483) who participated in a web-based survey about the ability to identify health misinformation on WhatsApp messages between November and December 2022.

			Values, n (%)
**Nationality**			
	Saudi	457 (94.6)	
	Non-Saudi	26 (5.4)	
**Age (years)**			
	18-24	130 (26.9)	
	25-34	173 (35.8)	
	35-44	99 (20.5)	
	45-54	47 (9.7)	
	55 or older	34 (7)	
**Sex**			
	Male	183 (37.9)	
	Female	300 (62.1)	
**Educational level**			
	High school and below	94 (19.4)	
	Diploma	39 (8.1)	
	Bachelor degree	275 (56.9)	
	Postgraduate degree	75 (15.5)	
**Employment status**			
	Student	114 (23.6)	
	Employed	220 (45.5)	
	Not employed	122 (25.3)	
	Retired	27 (5.6)	
**Region of residence**			
	East	181 (37.5)	
	Central	132 (27.3)	
	West	83 (17.2)	
	North	48 (9.9)	
	South	39 (8.1)	

Despite some variations, the sample matched the age and sex distribution of the Saudi Arabian population. Similarities in regional distribution between our sample and the populations of certain regions were also evident in the sample, with 27.3% (n=132) from the central region and 9.9% (n=48) from the northern region aligning with the national census in Saudi Arabia (n=5,365,700, 28.5% and n=1,877,108, 9.9%, respectively). In the eastern region, our sample showed a higher representation at 37.5% (n=181) compared with 15.7% (n=2,949,854) reported in the census data [40]. Our sample also showed a difference in educational level, with 56.9% (n=275) of participants holding bachelor degrees, in contrast to the national statistic of 23% (n=2,812,477) [41].

### Association Between Knowledge Level and Demographic Variables

The highest possible score for the survey questions was 8 points (correct answers were scored as 1 point; incorrect answers and responses of “I do not know” were scored as 0 points). We used a modified Bloom cutoff value of 75% (6 points) to categorize the participants’ knowledge. A knowledge score of ≥6 indicated a high level of knowledge, while a score of <6 indicated a low level of knowledge.

The associations between knowledge about identifying misinformation in WhatsApp messages and demographic variables were assessed using chi-square tests (Table 4). There were statistically significant associations between knowledge level and age, sex, employment, and region of residence (*P*<.05).

**Table 4:** Chi-square tests to examine the association between knowledge about identifying misinformation in WhatsApp messages before the intervention and demographic variables (N=483).

Factor		Low knowledge, n (%)	High knowledge, n (%)	Chi-square (*df*)		*P* value	
**Nationality**					0.529 (1)		.47
	Saudi	136 (29.8)	321 (70.2)				
	Non-Saudi	6 (23.1)	20 (76.9)				
**Age (years)**					43.030 (4)		<.001
	18-24	22 (16.9)	108 (83.1)				
	25-34	37 (21.4)	136 (78.6)				
	35-44	41 (41.4)	58 (58.6)				
	45-54	22 (46.8)	25 (53.2)				
	55 and above	20 (58.8)	14 (41.2)				
**Sex**					4.409 (1)		.04
	Male	64 (35)	119 (65)				
	Female	78 (26)	222 (74)				
**Education**					7.289 (3)		.06
	High school and below	35 (37.2)	59 (62.8)				
	Diploma	16 (41)	23 (59)				
	Bachelor degree	71 (25.8)	204 (74.2)				
	Postgraduate degree	20 (26.7)	55 (73.3)				
**Employment**					21.378 (3)		<.001
	Student	19 (16.7)	95 (83.3)				
	Employed	71 (32.3)	149 (67.7)				
	Not employed	36 (29.5)	86 (70.5)				
	Retired	16 (59.3)	11 (40.7)				
**Region of residence**					35.330 (4)		<.001
	East	61 (33.7)	120 (66.3)				
	Central	25 (18.9)	107 (81.1)				
	West	13 (15.7)	70 (84.3)				
	North	27 (56.3)	21 (43.8)				
	South	16 (41)	23 (59)				

### Effectiveness of the Intervention

The Kolmogorov-Smirnov test and the Shapiro-Wilk test indicated that the knowledge scores before and after the educational video were not normally distributed (*P*<.001). Since the distribution was not symmetric, it is negatively skewed, and there are a few outliers on the left side contributing to the skewness; nonparametric tests were used to assess statistical significance.

The median scores were assessed both before (median = 7, IQR = 5-8) and after (median = 8, IQR = 6-8) the video intervention, using the Wilcoxon signed-rank test. This comparison revealed that the video intervention did elicit a statistically significant change in the participants’ abilities to identify misinformation in WhatsApp messages (*z*=–6.887; *P*<.001).

### Knowledge Questions

The proportions of correct answers per individual test question before and after the video intervention were compared using the McNemar test. Significant differences in the participants’ pre- and postintervention knowledge about identifying misinformation were found for specific questions (Table 5). Viewing the video was associated with increased knowledge about the following concepts: checking the “forwarded” label (*P*<.001), looking for spelling and grammatical errors (*P*<.001), analyzing the facts (*P*=.03), checking links (*P*=.002, *P*=.001), and assessing the photos and videos (*P*<.001).

**Table 5:** Participants’ answers before and after viewing the educational video (N=483).

Domains and answer				Preintervention, n (%)		Postintervention, n (%)			*P* value
**Check the “Forwarded” label**									
	**Q1**							<.001	
		Correct	387 (80.1)		423 (87.6)				
		Incorrect	96 (19.9)		60 (12.4)				
**Look for spelling and grammatical errors**									
	**Q2**							<.001	
		Correct	357 (73.9)		402 (83.2)				
		Incorrect	126 (26.1)		81 (16.8)				
**Read beyond the headline**									
	**Q4**							.061	
		Correct	416 (86.1)		430 (89.0)				
		Incorrect	67 (13.9)		53 (11)				
**Analyze the facts**									
	**Q5**							.028	
		Correct	418 (86.5)		435 (90.1)				
		Incorrect	65 (13.5)		48 (9.9)				
**Check links**									
	**Q3**							.54	
		Correct	387 (80.1)		402 (83.2)				
		Incorrect	96 (19.9)		81 (16.8)				
	**Q6**							.002	
		Correct	326 (67.5)		354 (73.3)				
		Incorrect	157 (32.5)		129 (26.7)				
	**Q8**							.001	
		Correct	377 (78.1)		403 (83.4)				
		Incorrect	106 (21.9)		80 (16.6)				
**Assess the photos and videos**									
	**Q7**							<.001	
		Correct	333 (68.9)		368 (76.2)				
		Incorrect	150 (31.1)		115 (23.8)				

### Improvement in Knowledge Level

The health misinformation education intervention involved 483 participants. Pretest results showed that 70.6% (n=341) of participants had high knowledge (score ≥6), while 29.4% (n=142) had low knowledge (score>6). After the posttest, 10.6% (n=51) of the sample had improved to high knowledge and 3.3% (n=16) had lower scores, indicating 77.8% (n=376) had a score of 6 or above. McNemar test determined that there was a statistically significant difference in knowledge level before and after the intervention (*P*<.001; Table 6).

**Table 6:** McNamar test to compare knowledge level before and after the intervention (n=483; *P*<.001).

		After, n (%)	
		Low	High
**Before, n (%)**			
Low		91 (18.9)	51 (10.5)
High		16 (3.3)	325 (67.3)

## Discussion

### Overview

This study aimed to design and evaluate the effectiveness of an educational video to improve the abilities of participants in Saudi Arabia to identify health misinformation within the WhatsApp app. The study used a single-arm, pre-post intervention design and was conducted on the web. The effectiveness of the intervention was assessed. Furthermore, the participants’ knowledge levels about identifying misinformation were assessed before and after the intervention as were the associations between the participants’ characteristics and their knowledge.

The proliferation of health-related misinformation on social media has raised public health concerns in many countries [42]. Chen et al [43] found that people with limited health literacy were more likely to trust health-related information found on social media and blogs. Thus, improving the public’s ability to evaluate health information may be necessary. In Saudi Arabia, WhatsApp is the most popular social network, which is used by 87.4% (n=30.67 million) of internet users [6,24]. This platform has been identified as one on which misinformation may be easily spread [44]; therefore, it was the platform on which this study focused.

### Principal Results

The participants’ ability to identify misinformation in WhatsApp messages significantly improved following the educational intervention (*P*<.001). This result supports the finding of a systematic review by Cusack et al [13], which showed that, at least in the short term, educational interventions could improve knowledge and skills. This finding is also in line with the message interpretation process theory and the inoculation theory, in which interventions and prior messages are identified as factors that effectively protect against the harm caused by misinformation [19,21].

Additionally, the findings of this study suggest that literacy interventions combined with visual multimedia may improve misinformation detection. Apuke et al [45] found that participants who received visual multimedia education had better knowledge of literacy concepts than those who were educated without visual multimedia. Thus, as previously mentioned in this study, multimedia enhances memory, as stated in the cognitive theory of multimedia learning. The receiver’s exposure to the various message components makes it easier to integrate new information [23].

In this study, the items that assessed the following concepts (checking the “forwarded” label, looking for spelling and grammatical errors, analyzing the facts, assessing the photos and videos, and checking links) were significantly associated with improvements in the participants’ knowledge (*P*<.05 for all). However, the item related to the concept of reading beyond the headline was not significantly associated with improvement (*P*>.05).

It was noticed that WhatsApp messages with misinformation are characterized by requests to forward the message to many people. Further, many forwarded messages include fake information sources such as links or names and are vague about timelines, authors, and origins. Consequently, most forwarded messages are found to contain misinformation [46].

By recognizing the most prominent characteristics of health misinformation, users can improve their abilities to identify it on social media. Some studies have proposed criteria such as accuracy, authority, objectivity, and currency, but it is challenging for laypeople to evaluate these indicators. The role of such criteria is quite limited for general users, who, by definition, are not professionals [47]. Li et al [47] proposed a feature scheme and incorporated semantic, grammatical, and peripheral features of messages in evaluating their credibility. Their developed feature scheme allowed users to improve both their abilities to recognize health misinformation and their levels of digital health literacy.

This study found a significant association between knowledge level and age, sex, employment, and region of residence (*P*<.05 for all). Bapaye and Bapaye [48] noted that those engaged in elementary occupations and those older than 65 years of age were most likely to get false information from WhatsApp in a developing country. Workers in the health care industry were not immune from the impact of false information and were found to be just as susceptible as those in other professions.

The pretest results showed that 70.6% (n=341) of participants had good levels of knowledge about identifying misinformation on WhatsApp. There may have been factors contributing to an increase in knowledge, such as public awareness campaigns and government efforts. During the pandemic, the Saudi Arabian Ministry of Health conducted a comprehensive media campaign that included television, websites, and social media. Taking advantage of social media platforms, the Ministry of Health also engages with the public and the media. In addition to these early initiatives, efforts have been made to combat rumors and misinformation and engage the public in prevention and control measures [49].

Higher health literacy levels are associated with more favorable perceptions of health information. However, health literacy varies depending on the situation, and thus even those with high levels of health literacy may need help occasionally. For instance, those unfamiliar with medical language may find it challenging to distinguish between materials that provide accurate information and those with inaccurate information. Health care professionals and organizations must evaluate the population’s level of health literacy in order to ensure that people have access to adequate information when it matters most. Strategies like awareness-raising campaigns, community engagement, educational interventions, and training programs should be implemented when needed [50]. In accordance with the first Infodemiology Conference of the World Health Organization, public health authorities must create, evaluate, implement, and adapt tools and strategies for managing infodemics in acute public health crises in a manner that is suitable for their countries and situations [15]. This study’s findings may provide insight to public health authorities about developing an appropriate intervention for the population.

### Strengths and Limitations

The strength of this study is that it involved developing and validating an educational video in the Arabic language to identify misinformation on WhatsApp. Few Arabic educational materials exist to combat misinformation. The study’s limitations include some sampling bias due to the use of convenience sampling, which is a nonprobability sampling technique. While this technique may limit the generalizability of the results, it was appropriate for our study because it is more cost-effective, faster, and more direct than other sampling techniques.

### Conclusions

Health misinformation is an issue threatening public health because it dominates social media. Training people on the characteristics and practical applications of social media is urgently necessary. This study developed and evaluated the effectiveness of an educational video intervention to improve health misinformation identification on WhatsApp among the Saudi Arabian population. The results indicate that educational videos can be valuable tools for improving participants’ abilities to identify misinformation. The outcomes of this research can contribute to our understanding of effective tools for enhancing health misinformation awareness. These interventions can be particularly useful in combating misinformation in Arabic-speaking populations on WhatsApp, which may ultimately improve eHealth literacy. Limiting the prevalence and impact of misinformation allows people to make better-informed health care decisions. Our findings may also be helpful for health care professionals and organizations deciding on interventions suitable for providing access to adequate information to certain populations when needed.

## Appendix

Multimedia Appendix 1 The developed educational video (Health Misinformation Identification on WhatsApp).

Multimedia Appendix 2 Experts names and positions.

Multimedia Appendix 3 The video content validation form.

Multimedia Appendix 4 WhatsApp messages evaluation concepts and the corresponding survey questions used to assess them.

Multimedia Appendix 5 The final version of health misinformation identification survey.

## Abbreviations

I-CVI: item-level content validity index
S-CVI/Ave: scale-level content validity index averaging method
WHO: World Health Organization

## Data Availability

The data sets generated and analyzed during this study are available from the corresponding author on reasonable request.

## References

[1] Swire-Thompson B, Lazer D (2020). Public health and online misinformation: challenges and recommendations. Annu Rev Public Health.

[2] Dalmer NK (2017). Questioning reliability assessments of health information on social media. J Med Libr Assoc.

[3] Chou WYS, Gaysynsky A, Cappella JN (2020). Where we go from here: health misinformation on social media. Am J Public Health.

[4] Zielinski C (2021). Infodemics and infodemiology: a short history, a long future. Rev Panam Salud Publica.

[5] Greenspan RL, Loftus EF (2021). Pandemics and infodemics: research on the effects of misinformation on memory. Hum Behav Emerg Technol.

[6] Alshareef M, Alotiby A (2021). Prevalence and perception among Saudi Arabian population about resharing of information on social media regarding natural remedies as protective measures against COVID-19. Int J Gen Med.

[7] (2022). General Authority for Statistics: The Kingdom of Saudi Arabia.

[8] (2020). Survey of access and usage households and individuals to ICT 2020. General Authority for Statistics.

[9] Alasmari A, Addawood A, Nouh M, Rayes W, Al-Wabil A (2021). A retrospective analysis of the COVID-19 infodemic in Saudi Arabia. Future Internet.

[10] Tan EY, Wee RR, Saw YE, Heng KJ, Chin JW, Tong EM, Liu JC (2021). Tracking private WhatsApp discourse about COVID-19 in Singapore: longitudinal infodemiology study. J Med Internet Res.

[11] Isaakidou M, Diomidous M (2022). The contribution of informatics to overcoming the COVID-19 fake news outbreak by learning to navigate the infodemic. Stud Health Technol Inform.

[12] Song S, Zhang Y, Yu B (2021). Interventions to support consumer evaluation of online health information credibility: a scoping review. Int J Med Inform.

[13] Cusack L, Del Mar CB, Chalmers I, Hoffmann TC (2016). Educational interventions to improve people's understanding of key concepts in assessing the effects of health interventions: a systematic review protocol. Syst Rev.

[14] Di Sotto S, Viviani M (2022). Health misinformation detection in the social web: an overview and a data science approach. Int J Environ Res Public Health.

[15] Calleja N, AbdAllah A, Abad N, Ahmed N, Albarracin D, Altieri E, Anoko JN, Arcos R, Azlan AA, Bayer J, Bechmann A, Bezbaruah S, Briand SC, Brooks I, Bucci LM, Burzo S, Czerniak C, De Domenico M, Dunn AG, Ecker UKH, Espinosa L, Francois C, Gradon K, Gruzd A, Gülgün BS, Haydarov R, Hurley C, Astuti SI, Ishizumi A, Johnson N, Restrepo DJ, Kajimoto M, Koyuncu A, Kulkarni S, Lamichhane J, Lewis R, Mahajan A, Mandil A, McAweeney E, Messer M, Moy W, Ngamala PN, Nguyen T, Nunn M, Omer SB, Pagliari C, Patel P, Phuong L, Prybylski D, Rashidian A, Rempel E, Rubinelli S, Sacco P, Schneider A, Shu K, Smith M, Sufehmi H, Tangcharoensathien V, Terry R, Thacker N, Trewinnard T, Turner S, Tworek H, Uakkas S, Vraga E, Wardle C, Wasserman H, Wilhelm E, Würz A, Yau B, Zhou L, Purnat TD (2021). A public health research agenda for managing infodemics: methods and results of the first WHO Infodemiology Conference. JMIR Infodemiology.

[16] Pian W, Chi J, Ma F (2021). The causes, impacts and countermeasures of COVID-19 "infodemic": a systematic review using narrative synthesis. Inf Process Manag.

[17] Diviani N, van den Putte B, Giani S, van Weert JC (2015). Low health literacy and evaluation of online health information: a systematic review of the literature. J Med Internet Res.

[18] Oh HJ, Lee H (2019). When do people verify and share health rumors on social media? The effects of message importance, health anxiety, and health literacy. J Health Commun.

[19] McGuire WJ, Haaland CC, Kaelber WO (1981). Inducing resistance to persuasion. Some contemporary approaches. Self and Society: An Anthology of Readings.

[20] Jeong SH, Cho H, Hwang Y (2012). Media literacy interventions: a meta-analytic review. J Commun.

[21] Austin EW, Gall SB, Arnet JJ (2007). Message Interpretation Process Model. Encyclopedia of Children, Adolescents, and the Media.

[22] Apuke OD, Omar B, Tunca EA (2022). Literacy concepts as an intervention strategy for improving fake news knowledge, detection skills, and curtailing the tendency to share fake news in Nigeria. Child Youth Serv.

[23] Mayer RE, Moreno R (2010). Nine ways to reduce cognitive load in multimedia learning. Educ Psychol.

[24] (2023). Saudi Arabia social media statistics 2023. Global Media Insight.

[25] (2022). Sample size calculator. Raosoft.

[26] Stratton SJ (2021). Population research: convenience sampling strategies. Prehosp Disaster Med.

[27] (2022). How to prevent the spread of misinformation. WhatsApp.

[28] Let's flatten the infodemic curve. World Health Organization.

[29] Wichowski DE, Kohl LE, Folk M, Apostel S (2012). Establishing credibility in the information jungle: blogs, microblogs, and the CRAAP test. Online Credibility and Digital Ethos: Evaluating Computer-Mediated Communication.

[30] Hugo J (1996). Prioritizing guidelines for health education message design. J Audiov Media Med.

[31] Yusoff MSB (2019). ABC of content validation and content validity index calculation. Educ Med J.

[32] de Sá Leite S, Áfio ACE, de Carvalho LV, da Silva JM, de Almeida PC, Pagliuca LMF (2018). Construction and validation of an educational content validation instrument in health. Rev Bras Enferm.

[33] da Rosa BVC, Girardon-Perlini NMO, Gamboa NSG, Nietsche EA, Beuter M, Dalmolin A (2019). Development and validation of audiovisual educational technology for families and people with colostomy by cancer. Texto Contexto Enferm.

[34] Hamid MRA, Yusof NDBM, Buhari SS, Malek KA, Noor HM (2021). Development and validation of educational video content, endorsing dietary adjustments among patients diagnosed with hypertension. Int J Health Promot Educ.

[35] Lynn MR (1986). Determination and quantification of content validity. Nurs Res.

[36] Polit DF, Beck CT (2006). The content validity index: are you sure you know what's being reported? Critique and recommendations. Res Nurs Health.

[37] Kingdom of Saudi Arabia—Ministry of Health Portal.

[38] Okello G, Izudi J, Teguzirigwa S, Kakinda A, Van Hal G (2020). Findings of a cross-sectional survey on knowledge, attitudes, and practices about COVID-19 in Uganda: implications for public health prevention and control measures. Biomed Res Int.

[39] Wahidiyat PA, Yo EC, Wildani MM, Triatmono VR, Yosia M (2021). Cross-sectional study on knowledge, attitude and practice towards thalassaemia among Indonesian youth. BMJ Open.

[40] Saudi census-population size. General Authority for Statistics.

[41] (2021). Demographic research bulletin 2016. General Authority for Statistics.

[42] Larson HJ (2018). The biggest pandemic risk? Viral misinformation. Nature.

[43] Chen X, Hay JL, Waters EA, Kiviniemi MT, Biddle C, Schofield E, Li Y, Kaphingst K, Orom H (2018). Health literacy and use and trust in health information. J Health Commun.

[44] Bowles J, Larreguy H, Liu S (2020). Countering misinformation via WhatsApp: preliminary evidence from the COVID-19 pandemic in Zimbabwe. PLoS One.

[45] Apuke OD, Omar B, Tunca EA, Gever CV (2022). The effect of visual multimedia instructions against fake news spread: a quasi-experimental study with Nigerian students. J Librariansh Inf Sci.

[46] Durul SS (2020). (Mis)information in baby boomers' WhatsApp messages. https://www.researchgate.net/publication/350522714_MISINFORMATION_IN_BABY_BOOMERS%27_WHATSAPP_MESSAGES.

[47] Li Y, Fan Z, Yuan X, Zhang X (2022). Recognizing fake information through a developed feature scheme: a user study of health misinformation on social media in China. Inf Process Manag.

[48] Bapaye JA, Bapaye HA (2021). Demographic factors influencing the impact of coronavirus-related misinformation on WhatsApp: cross-sectional questionnaire study. JMIR Public Health Surveill.

[49] (2020). WHO, Saudi Arabia join forces to fight COVID-19 nationally, regionally and globally. World Health Organization.

[50] Hange N, Agoli AM, Pormento MKL, Sharma A, Somagutta MR, Paikkattil N, Jadhav A, Bethineedi D, Pisude P (2022). Impact of COVID-19 response on public health literacy and communication. Health Promot Perspect.

