# Supplementary material for: Educational Video Intervention to Improve Health Misinformation Identification on WhatsApp Among Saudi Arabian Population: Pre-Post Intervention Study
Source: JMIR Form Res. 2024 Jan 17;8:e50211. doi: 10.2196/50211 (PMC10831668; doi:10.2196/50211)
Supplement: Multimedia Appendix 2 [file formative_v8i1e50211_app2.docx]

Expert names and position

|  | **Name** | **Position** |
| --- | --- | --- |
| 1 | Prof. Mowafa Househ | Professor of Digital Health, Hamad Bin Khalifa University, College of Science and Engineering, Qatar Foundation, Qatar. |
| 2 | Dr. Ranyah Aldekhyyel | Assistant Professor, Medical Informatics, King Saud University. |
| 3 | Dr. Mariam F. Alkazemi | Research assistant professor of public relations at Virginia Commonwealth University. |
| 4 | Dr. Khulud Alkadi | Assistant Professor, College of Public Health and Health Informatics, King Saud bin Abdul-Aziz University for Health Science. |
| 5 | Ms. Rokayya Konsowa | Risk Communication and Infodemic Management Officer, WHO Egypt. |
| 6 | Ms. Aseel Alshathri | Public Health Informatics Specialist at MOH |
| 7 | Ms. Bushra Alqarni | Senior Health Educator at Security Forces Hospital / Lecturer at King Saud University. |
