# Supplementary material for: Educational Video Intervention to Improve Health Misinformation Identification on WhatsApp Among Saudi Arabian Population: Pre-Post Intervention Study
Source: JMIR Form Res. 2024 Jan 17;8:e50211. doi: 10.2196/50211 (PMC10831668; doi:10.2196/50211)
Supplement: Multimedia Appendix 3 [file formative_v8i1e50211_app3.docx]

The video content validation form.

|  | **Objectives** | Disagree | Partially Agree | Agree |
| --- | --- | --- | --- | --- |
| 1 | The video's purpose is clear |  |  |  |
| 2 | It influences adopting new behaviors |  |  |  |
| 3 | It enables the clarification of doubts |  |  |  |
| 4 | It contributes to educational materials in the area |  |  |  |
| 5 | It stimulates interest in the theme |  |  |  |
|  | **Structure and Presentation** |  |  |  |
| 6 | The language is compatible with the public's understanding |  |  |  |
| 7 | It makes an objective presentation of the content |  |  |  |
| 8 | It clearly presents the content |  |  |  |
| 9 | The content shown is necessary |  |  |  |
| 10 | The ideas are exposed in a logical way |  |  |  |
| 11 | The theme is current |  |  |  |
| 12 | The size is adequate |  |  |  |
|  | **Audiovisual** |  |  |  |
| 13 | The timing is adequate |  |  |  |
| 14 | The illustrations are expressive and sufficient |  |  |  |
| 15 | The tone is friendly |  |  |  |
| 16 | There is an association between each scene's theme and the corresponding text |  |  |  |
| 17 | The characters/images are appropriate for the target audience |  |  |  |
