# Supplementary material for: Educational Video Intervention to Improve Health Misinformation Identification on WhatsApp Among Saudi Arabian Population: Pre-Post Intervention Study
Source: JMIR Form Res. 2024 Jan 17;8:e50211. doi: 10.2196/50211 (PMC10831668; doi:10.2196/50211)
Supplement: Multimedia Appendix 4 [file formative_v8i1e50211_app4.docx]

**WhatsApp messages evaluation concepts and the corresponding survey questions used to assess them**

| Concepts | Question |
| --- | --- |
| **Checking the "forwarded" label:**  **انتبه لعلامة "التحويل":**  عند إعادة توجيه رسالة معينة مرات عديدة، يتم تمييز هذه الرسالة بأيقونة سهمين مزدوجين. غالبًا ما تنتشر المعلومات غير الصحيحة انتشارًا سريعًا جدًا. تأكد من المعلومات الواردة في الرسالة عندما لا تكون على علم بكاتبها الأصلي.  **Pay attention to the "farwarded label":**  When a particular message is forwarded too many times, that message is marked with a double arrow icon. Incorrect information often spreads very quickly. Verify the information in the message when you do not know the original author.  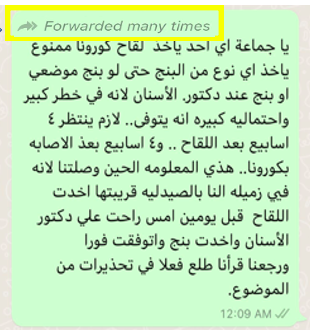  Forwarded many times  Hey people, anyone who takes corona vaccine is forbidden to take any kind of anesthesia, even if it is a local anesthetic or anesthesia at the den.tist, because he is in great danger and the possibility is high that he will die.. he must wait 4 weeks after the vaccine.. and 4 weeks afetr infection with corona.. just now we received this information because there was a_relative to a colleague of ours in the pharmacy took the vaccine two days ago. Yesterday, she went to the dentist and took anesthesia and immediately deied And we went back and read that there were actually warnings about the subject | Q 1:  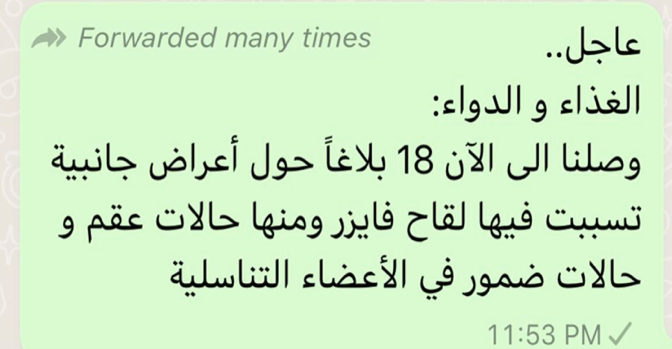  Forwarded many times  urgent..  Food and Drug:  We have so far reached 18 reports about side effects caused by the Pfizer vaccine, including cases of infertility and cases of atrophy in the genitals.  11:53 PM |
| **Looking for spelling and grammatical errors:**  **ابحث عن الأخطاء الإملائية أو النحوية:**  فكثير من الإشاعات لا ينتبه صاحبها لتلك الأمور والمصادر الرسمية لا تحتوي على هذه الأخطاء.  **Look for spelling or grammatical errors:**  Many rumors their owners do not pay attention to these matters, and the official sources do not contain these errors.  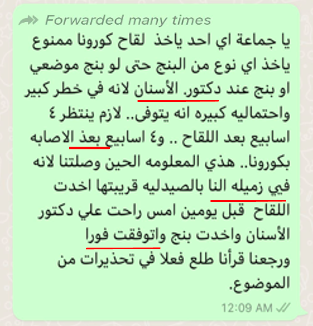  Forwarded many times  Hey people, anyone who takes corona vaccine is forbidden to take any kind of anesthesia, even if it is a local anesthetic or anesthesia at the den.tist, because he is in great danger and the possibility is high that he will die.. he must wait 4 weeks after the vaccine.. and 4 weeks afetr infection with corona.. just now we received this information because there was a_relative to a colleague of ours in the pharmacy took the vaccine two days ago. Yesterday, she went to the dentist and took anesthesia and immediately deied  And we went back and read that there were actually warnings about the subject | Q 2:  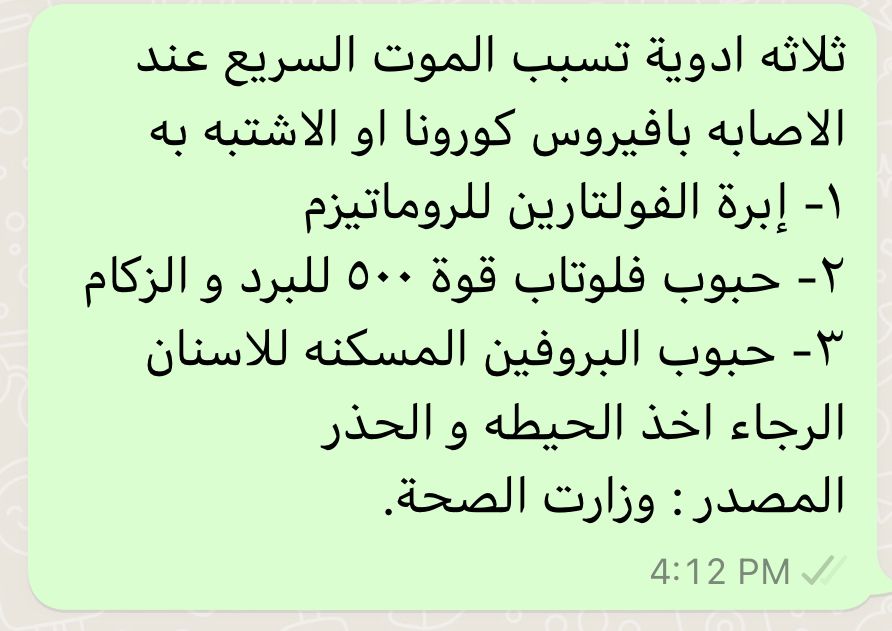 Three drugs that cause rapid death when infected with the Corona virus or suspected  1- Vottaren needle for rheumatism  2- Flutab pills of 500 power for cold and flu  3- Ibuprofen painkiller pills for teeth  Please take caution.  Source: Menistry of Health  4:12 PM |
| **Reading beyond the headline:**  **لا تكتفِ بقراءة العنوان الرئيسي فقط:**  قم بقراءة الرسالة كاملة. فبعض العناوين تكتب بشكل مثير او استفزازي لكي يتم مشاركتها لأكبر عدد ممكن.  **Don't just read the headline:**  Read the full message. Some titles are written in an exciting or provocative way in order to be shared to as many people as possible  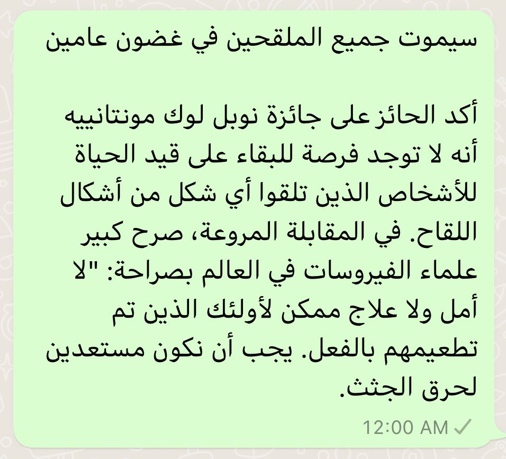 All vaccinated will die within two years  Nobel laureate Luc Montagnier emphasized that there is no chance of survival for people who have received any form of the vaccine. In the harrowing interview, the world's chief virologist bluntly states: "There is no hope and no cure possible for those who have already been vaccinated. We must be prepared to cremation. | Q 4:  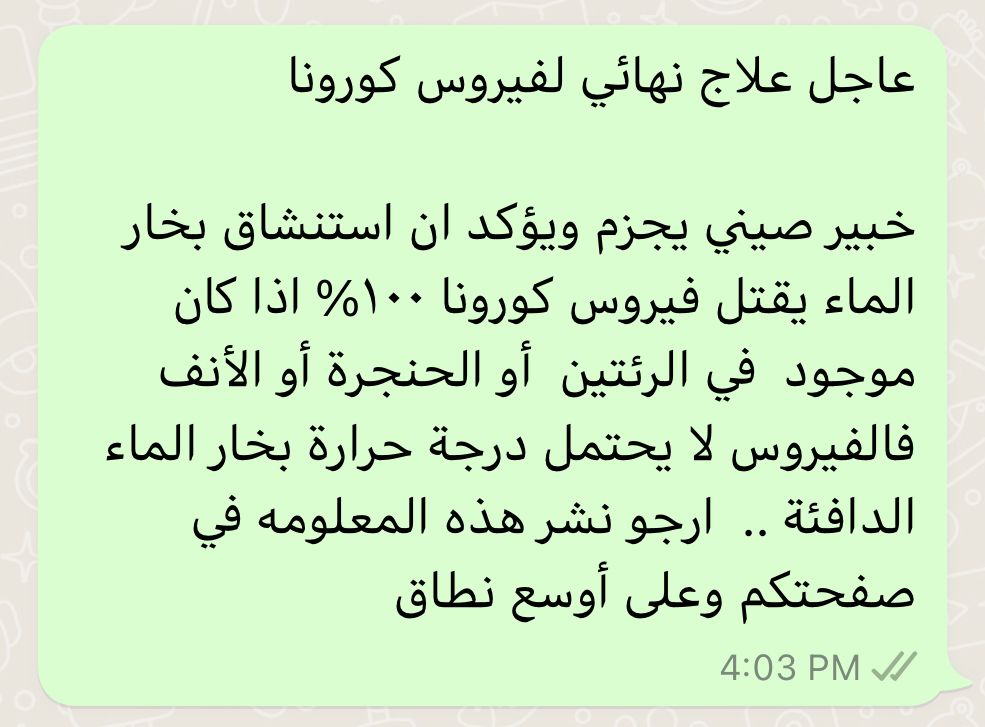 Urgent final treatment for corona virus  A Chinese expert asserts and confirms that inhaling water vapor kills the Corona virus 100% if it is present in the lungs, throat, or nose, as the virus cannot tolerate the warm temperature of the water vapor.. Please spread this information on your page and on the widest scale  4:03 PM |
| **Analyzing the facts:**  **حلل الحقائق:**  إذا كان الموضوع غير قابل للتصديق فهو بالغالب كذلك.  **Analyze the facts:**  if the subject is not believable, it probably is.  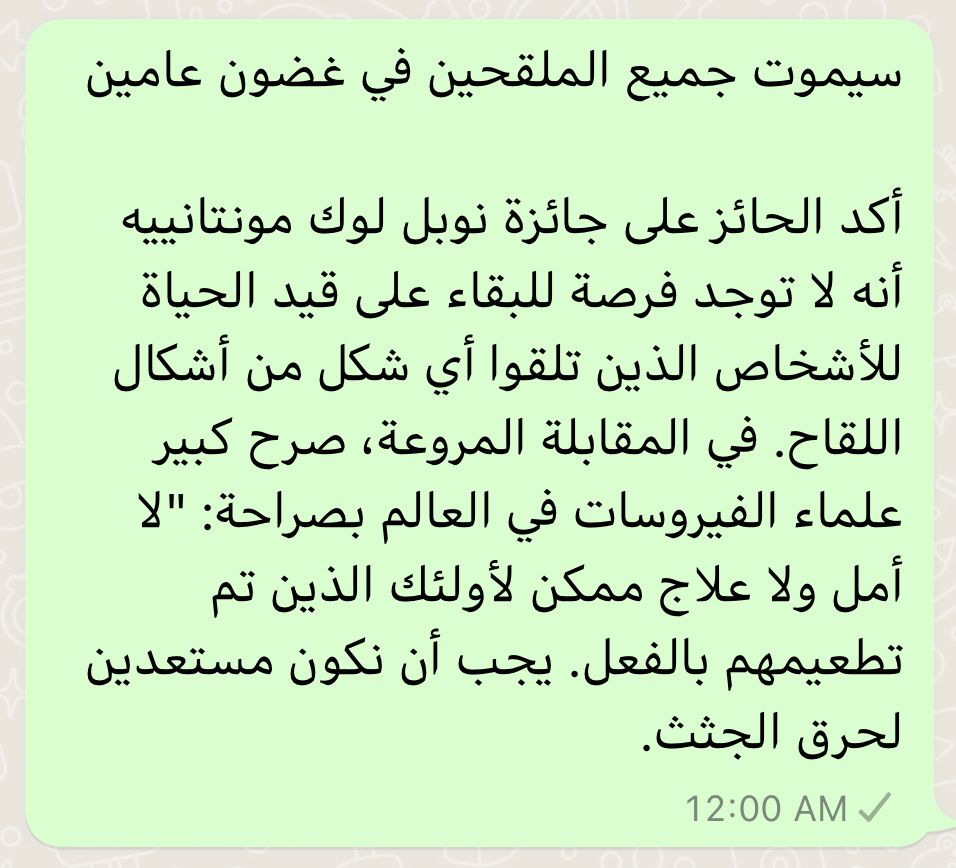 All vaccinated will die within two years  Nobel laureate Luc Montagnier emphasized that there is no chance of survival for people who have received any form of the vaccine. In the harrowing interview, the world's chief virologist bluntly states: "There is no hope and no cure possible for those who have already been vaccinated. We must be prepared to cremation. | Q 5:  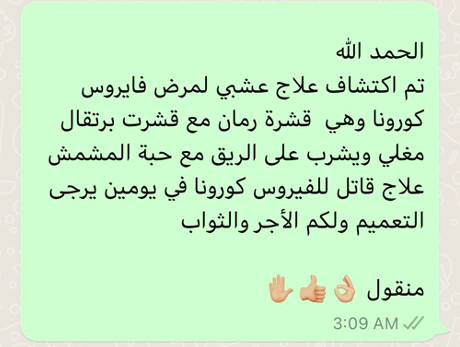  Praise be to God!  A herbal remedy for Corona virus disease has been discovered, which is a pomegranate peel with a boiled orange peel, and it is drunk on an empty stomach with an apiicot seed. A deadly treatment for the Corona virus in two days. Please circulate, and you will be rewarded  Quoted  3:09 AM |
| **Checking Links:**  **تحقق من الروابط:**  تأكد مما إذا كان الرابط يطابق المصدر أم لا، فمعظم المواقع الموثوقة تحتوي على gov. أو edu.  **Check out the links:**  Check if the link matches the source or not, most trusted sites contain .gov or edu.  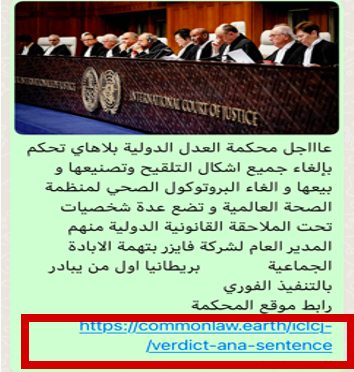  Urgent, the International Court of Justice in The Hague rules to cancel all forms of vaccination, manufacturing and selling it, and cancels the health protocol of the World Health Organization, and puts several personalities under international legal prosecution, including the General Manager of Pfizer on charges of genocide, with immediate implementation. Britain is the first to initiate Court website link https://cornmonlaw.earth/icici -/verdict-an a-sentence | Q (3, 6, 8)  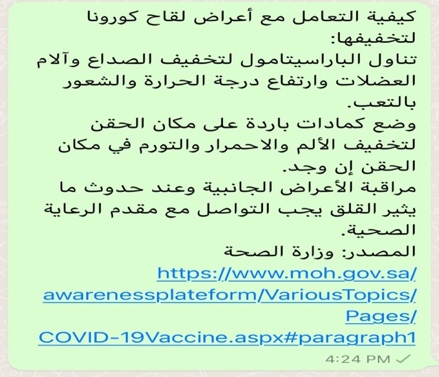  How to deal with the symptoms of the Corona vaccine to reduce it:  Take paracetamol to relieve headaches, muscle pain, high fever and tiredness.  Put cold compresses on the injection place to reduce pain, redness and swelling at the injection place, if any.  Monitor the side effects, and when a concern occurs, contact the health care provider.  Source: Ministry of Health https://www.moh.gov.sajawarenessplateform/VariousTopicsiPages/C0 VID-19Vaccine.aspx#paragraph1  4:24 PM  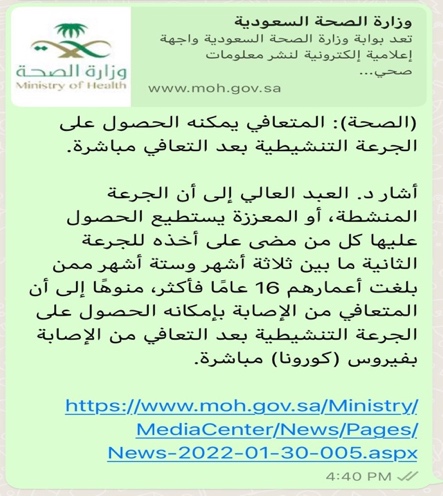  (Health): The recovered can take the booster dose immediately after recovery.  Dr. Al-Abd Al-Aali indicated indicated that the stimulant or booster dose can be taken by everyone who has taken the second dose between three and six months ago, whose aged 16 years and over, noting that the recovering from infection can take the booster dose after recovering from infection with the Corona virus directly.  https://www.moh.gov.saiMinistrv/MediaCenteriNews/PagesiNew-2- 2022-01-30-005.aspx  4:40 PM  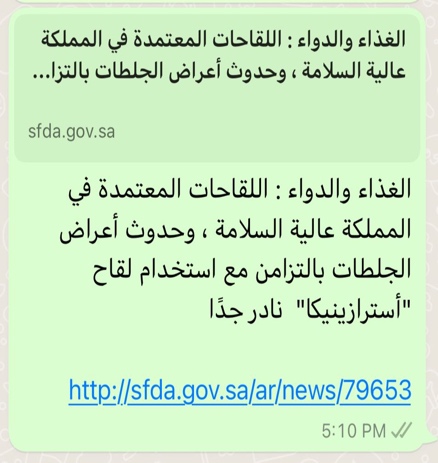  Food and Drug: Vaccines approved in the Kingdom are of high safety, and the occurrence of stroke symptoms in conjunction with the use of the "AstraZeneca" vaccine is very rare.  http://sfda.gov.satarinews/79653  5:10 PM |
| **Assessing the photos and videos:**  **تحقق من الصور والفيديوهات:**  تحقق من عدم وجود تعديلات على الصور والفيديوهات. من الممكن ان تكون الصور والفيديوهات صحيحة لكنها قديمة أو قد تكون القصة حولها مفبركة.  **Check out the photos and videos:**  Check that there are no modifications to the photos and videos. The pictures and videos may be true but old, or the story about them may be fabricated.  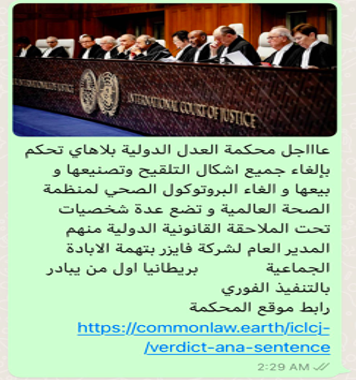  Urgent, the International Court of Justice in The Hague rules to cancel all forms of vaccination, manufacturing and selling it, and cancels the health protocol of the World Health Organization, and puts several personalities under international legal prosecution, including the General Manager of Pfizer on charges of genocide, with immediate implementation. Britain is the first to initiate Court website link https://cornmonlaw.earth/icici -/verdict-an a-sentence | 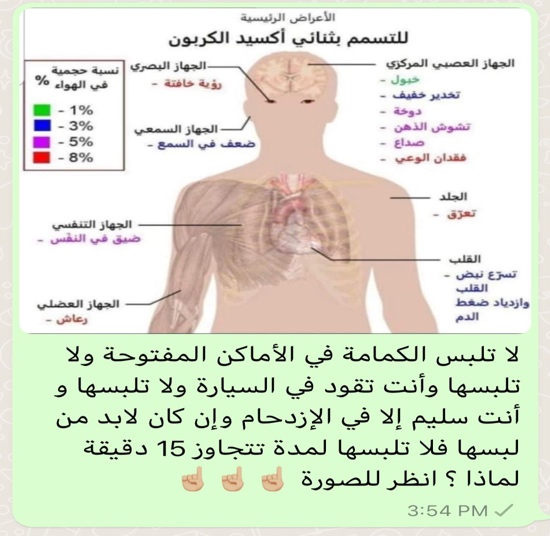  Q 7:  Do not wear the mask in open places, do not wear it while you are driving in the car, and do not wear it while you are intact, except in the crowding, and if it is necessary to wear it, do not wear it for more than 15 minutes. Why? See the picture  3:54 PM |
