# Supplementary material for: Educational Video Intervention to Improve Health Misinformation Identification on WhatsApp Among Saudi Arabian Population: Pre-Post Intervention Study
Source: JMIR Form Res. 2024 Jan 17;8:e50211. doi: 10.2196/50211 (PMC10831668; doi:10.2196/50211)
Supplement: Multimedia Appendix 5 [file formative_v8i1e50211_app5.docx]

Survey:

| المملكة العربية السعودية  kingdom of Saudi Arabia  جامعة الملك سعود بن عبد العزيز للعلوم الصحية  King Saud bin Abdulaziz University for Health Sciences  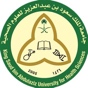 | | |
| --- | --- | --- |
| السلام عليكم ورحمة الله وبركاته  أنت مدعو للانضمام طواعية لدارسة بحثية لقياس مدى قدرة المشاركين على تمييز رسائل الواتساب التي تحتوي على المعلومات الصحية المغلوطة.  الباحثة: ابتهال السعد  هذه الدراسة تهدف إلى تطوير مادة تعليمية لتثقيف المجتمع حول كيفية تحديد المعلومات الخاطئة المتعلقة بالصحة في رسائل الواتساب .(WhatsApp) و لتقييم فعالية المادة التعليمية في تثقيف المجتمع حول تحديد المعلومات الخاطئة المتعلقة بالصحة على الواتساب (WhatsApp)  إن مشاركتك في هذه الدراسة طوعية ولك الحق التام في عدم قبول تعبئة الاستبيان أو الانسحاب في أي وقت تشاء بدون ابداء الأسباب.  هذا الاستبيان جزء من بحث تخرج طالبة ماجستير، تخصص المعلوماتية الصحية في جامعة الملك سعود بن عبد العزيز للعلوم الصحية. إكمالك الاستبيان تعني الموافقة على استخدام المعلومات لأغراض علمية وستحتفظ البيانات بسريتها ولن يتم استخدامها سوى لأغراض الدراسة والبحث العلمي.  (E-mail: Alsaad32004@ksau-hs.edu.sa) إذا كان لديك أي اسئلة حول هذا البحث ، يرجى التواصل عبر البريد الإلكتروني  في حال كان لديك الاستفسارات المتعلقة بحقوقك كموضوع بحث يمكنك الاتصال بمجلس المراجعة المؤسسية على هاتف 8011111 تحويلة 14572  May peace, blessings, and mercy of God be upon You are invited to voluntarily join a research recognize WhatsApp messages containing health  Researcher: Ebtihal AlSaad.  This study aims to develop an educational material to educate the community about how to identify health-related misinformation in WhatsApp messages and to evaluate the effectiveness of the educational material in educating the community about identifying health-related misinformation on WhatsApp. Your participation in this study is voluntary, and you have the full right not to accept filling out the questionnaire or to withdraw at any time without giving reasons.  This questionnaire is part of the graduate research of a master's student majoring in health informatics at King Saud bin Abdulaziz University for Health Sciences. By completing the questionnaire, you agree to use the information for scientific purposes, and the data will remain confidential and will only be used for the purposes of the study and scientific research.  If you have any questions about this research, please contact us via e-mail: (Alsaad32004@ksau-hs.edu.sa). In the event that you have inquiries regarding your rights as a research subject, you can contact the Institutional Review Board at Tel.No.: 8011111 Ext. No.: 14572 | | |
| الجنس: (Gender) ذكر (Male) انثى (Female) | | ١ |
| العمر: (Age) ١٨-٢٩ (18-19) ٢٥-٣٤ (25-34) ٣٥-٤٤ (35-44)    ٤٥-٥٤(45-54) ٥٥ وأكبر (55 and over) | | ٢ |
| المستوى التعليمي: غير متعلم ابتدائي  )Intermediate(  متوسط  )Elementary(  (Uneducated)    (Educational Level)  (Bachelor)  بكالوريوس  (Diploma)  دبلوم  )Secondary(  ثانوي  (Postgraduate)  دراسات عليا | | ٣ |
| العمل: طالب  متقاعد (Retired)  غير موظف (Unemployed)  موظف (Employed)  (Student)  (Occupation) | | ٤ |
| منطقة الإقامة: المنطقة الشرقية  المنطقة الغربية (Western Region)  المنطقة الجنوبية (Southern Region)  المنطقة الشمالية (Northern Region)  المنطقة الوسطى (Central Region)  (Eastern Region)  (Area of Residence) | | ٥ |
| مدينة الإقامة: ................ | | ٦  (City of Residence) |
| الجنسية: سعودي  (Saudi)  (Non-Saudi (Resident)) غير سعودي (مقيم)  (Nationality) | | ٧ |
| ١- ما هو تقييمك لهذه الرسالة:  (What is your assessment of this message:)  (Correct)  صحيحة  (False)  خاطئة  (I don’t know)  لا أعلم | 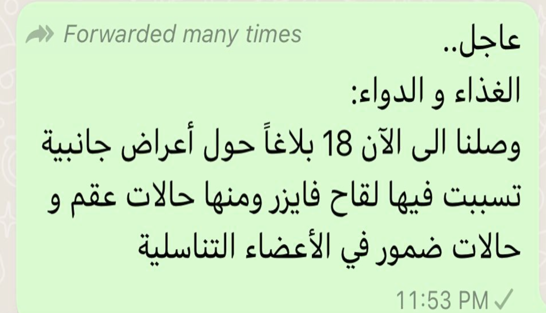  Forwarded many times  urgent..  Food and Drug:  We have so far reached 18 reports about side effects caused by the Pfizer vaccine, including cases of infertility and cases of atrophy in the genitals.  11:53 PM | |
| ٢- ما هو تقييمك لهذه الرسالة:  (What is your assessment of this message:)  (Correct)  صحيحة  (False)  خاطئة  (I don’t know)  لا أعلم | 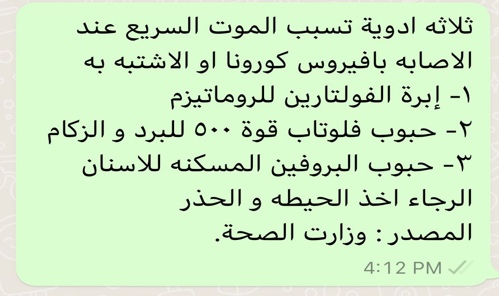  Three drugs that cause rapid death when infected with the Corona virus or suspected  1- Vottaren needle for rheumatism  2- Flutab pills of 500 power for cold and flu  3- Ibuprofen painkiller pills for teeth  Please take caution.  Source: Menistry of Health  4:12 PM | |
| ٣- ما هو تقييمك لهذه الرسالة:  (What is your assessment of this message:)  (Correct)  صحيحة  (False)  خاطئة  (I don’t know)  لا أعلم | 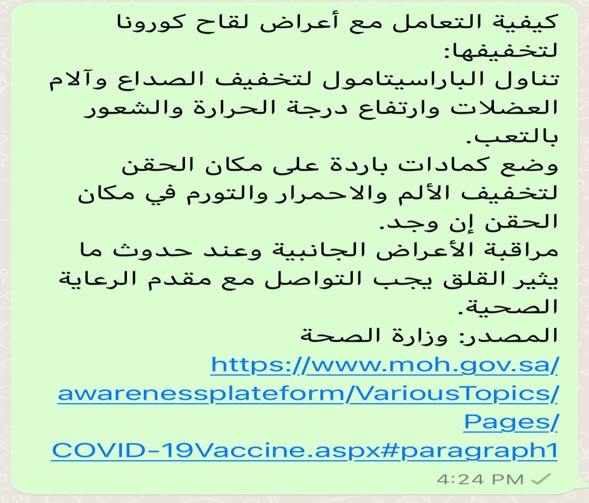  How to deal with the symptoms of the Corona vaccine to reduce it:  Take paracetamol to relieve headaches, muscle pain, high fever and tiredness.  Put cold compresses on the injection place to reduce pain, redness and swelling at the injection place, if any.  Monitor the side effects, and when a concern occurs, contact the health care provider.  Source: Ministry of Health https://www.moh.gov.sajawarenessplateform/VariousTopicsiPages/C0 VID-19Vaccine.aspx#paragraph1  4:24 PM | |
| ٤- ما هو تقييمك لهذه الرسالة:  (What is your assessment of this message:)  (Correct)  صحيحة  (False)  خاطئة  (I don’t know)  لا أعلم | 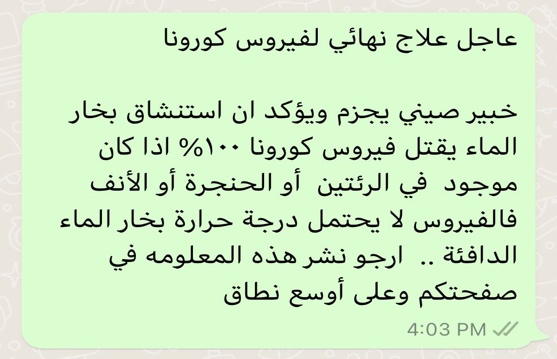  Urgent final treatment for corona virus  A Chinese expert asserts and confirms that inhaling water vapor kills the Corona virus 100% if it is present in the lungs, throat, or nose, as the virus cannot tolerate the warm temperature of the water vapor.. Please spread this information on your page and on the widest scale  4:03 PM | |
| ٥- ما هو تقييمك لهذه الرسالة:  (What is your assessment of this message:)  (Correct)  صحيحة  (False)  خاطئة  (I don’t know)  لا أعلم | 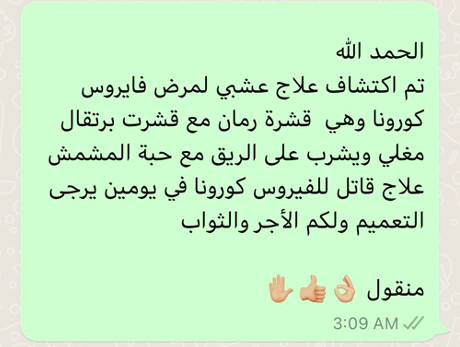  Praise be to God!  A herbal remedy for Corona virus disease has been discovered, which is a pomegranate peel with a boiled orange peel, and it is drunk on an empty stomach with an apiicot seed. A deadly treatment for the Corona virus in two days. Please circulate, and you will be rewarded  Quoted  3:09 AM | |
| ٦- ما هو تقييمك لهذه الرسالة:  (What is your assessment of this message:)  (Correct)  صحيحة  (False)  خاطئة  (I don’t know)  لا أعلم | 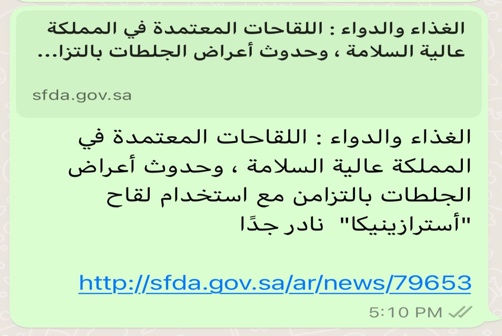  Food and Drug: Vaccines approved in the Kingdom are of high safety, and the occurrence of stroke symptoms in conjunction with the use of the "AstraZeneca" vaccine is very rare.  http://sfda.gov.satarinews/79653  5:10 PM | |
| ٧- ما هو تقييمك لهذه الرسالة:  (What is your assessment of this message:)  (Correct)  صحيحة  (False)  خاطئة  (I don’t know)  لا أعلم | 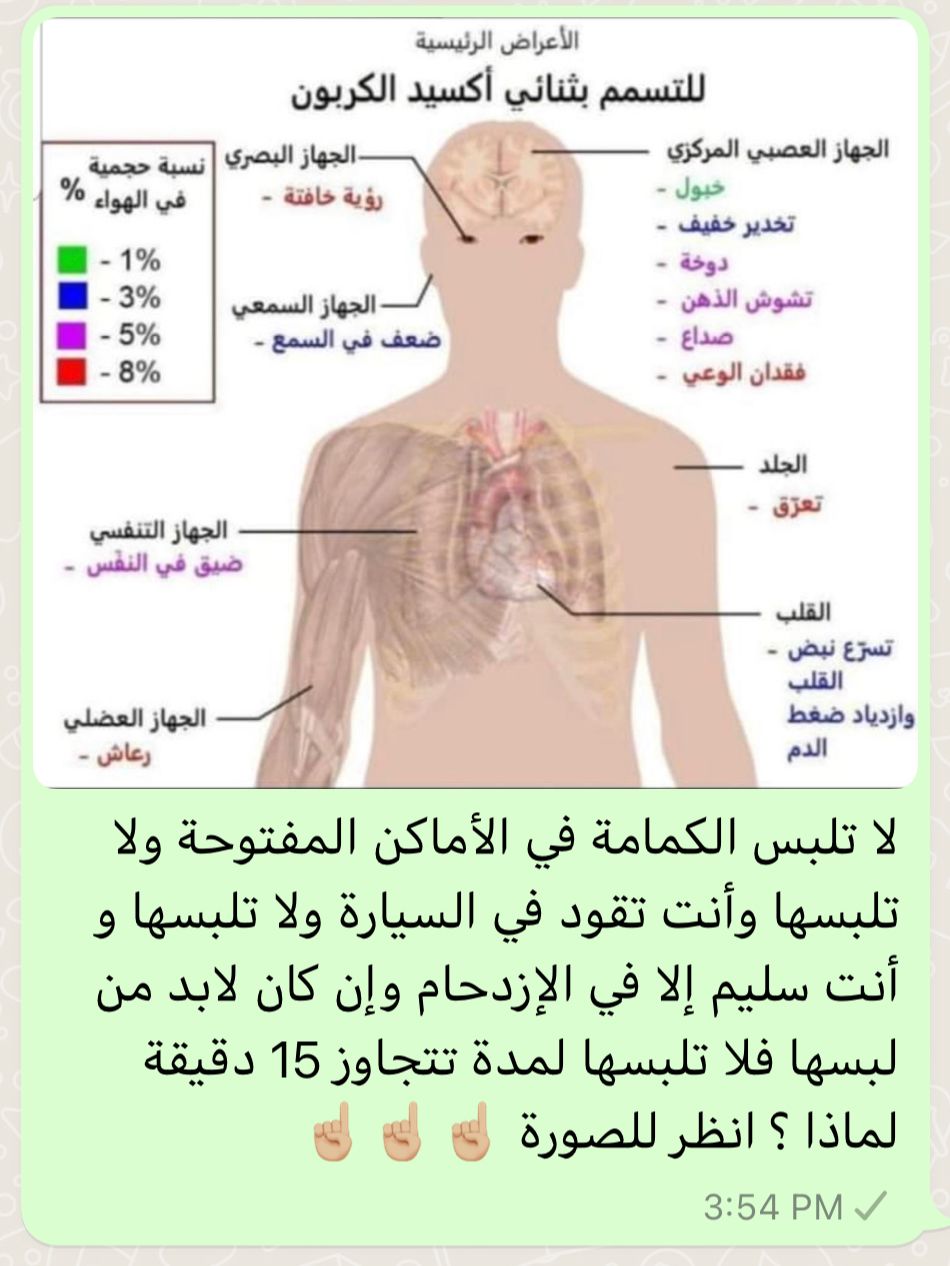  Do not wear the mask in open places, do not wear it while you are driving in the car, and do not wear it while you are intact, except in the crowding, and if it is necessary to wear it, do not wear it for more than 15 minutes. Why? See the picture  3:54 PM | |
| ٨- ما هو تقييمك لهذه الرسالة:  (What is your assessment of this message:)  (Correct)  صحيحة  (False)  خاطئة  (I don’t know)  لا أعلم | 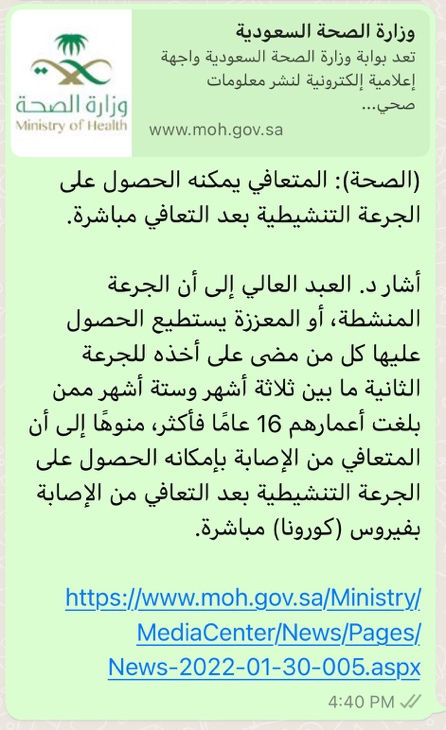  (Health): The recovered can take the booster dose immediately after recovery.  Dr. Al-Abd Al-Aali indicated indicated that the stimulant or booster dose can be taken by everyone who has taken the second dose between three and six months ago, whose aged 16 years and over, noting that the recovering from infection can take the booster dose after recovering from infection with the Corona virus directly.  https://www.moh.gov.saiMinistrv/MediaCenteriNews/PagesiNew-2- 2022-01-30-005.aspx  4:40 PM | |
